# Antibody responses after COVID-19 infection in patients who are mildly symptomatic or asymptomatic in Bangladesh

**DOI:** 10.1016/j.ijid.2020.09.1484

**Published:** 2020-12

**Authors:** Tahmina Shirin, Taufiqur Rahman Bhuiyan, Richelle C. Charles, Shaheena Amin, Imran Bhuiyan, Zannat Kawser, Asifuzaman Rahat, Ahmed Nawsher Alam, Sharmin Sultana, Md Abdul Aleem, Manjur Hossain Khan, Samsad Rabbani Khan, Regina C. LaRocque, Stephen B. Calderwood, Edward T. Ryan, Damien M. Slater, Sayera Banu, John Clemens, Jason B. Harris, Meerjady Sabrina Flora, Firdausi Qadri

**Affiliations:** a Institute of Epidemiology, Disease Control and Research, Dhaka, Bangladesh; b Infectious Diseases Division, icddr,b, Mohakhali, Dhaka, Bangladesh; c Division of Infectious Diseases, Massachusetts General Hospital, Boston, MA, USA; d Departments of Medicine and Pediatrics, Harvard Medical School, Boston, MA, USA; e Institute for Developing Science & Health Initiatives (ideSHi), Dhaka, Bangladesh; f Department of Immunology and Infectious Diseases, Harvard T.H. Chan School of Public Health, Boston, MA, USA

**Keywords:** Seroconversion, COVID-19, Immune responses, Symptomatic, Asymptomatic

## Abstract

**Objectives:**

Studies on serological responses following coronavirus disease-2019 (COVID-19) have been published primarily in individuals who are moderately or severely symptomatic, but there are few data from individuals who are mildly symptomatic or asymptomatic.

**Methods:**

We measured IgG, IgM, and IgA to the receptor-binding domain of severe acute respiratory syndrome coronavirus 2 (SARS-CoV-2) by using enzyme-linked immunosorbent assay in mildly symptomatic (n = 108) and asymptomatic (n = 63) on days 1, 7, 14, and 30 following RT-PCR confirmation in Bangladesh and when compared with pre-pandemic samples, including healthy controls (n = 73) and individuals infected with other viruses (n = 79).

**Results:**

Mildly symptomatic individuals developed IgM and IgA responses by day 14 in 72% and 83% of individuals, respectively, while 95% of individuals developed IgG response, and rose to 100% by day 30. In contrast, individuals infected with SARS-CoV-2 but who remained asymptomatic developed antibody responses significantly less frequently, with only 20% positive for IgA and 22% positive for IgM by day 14, and 45% positive for IgG by day 30 after infection.

**Conclusions:**

These results confirm immune responses are generated following COVID-19 who develop mildly symptomatic illness. However, those with asymptomatic infection do not respond or have lower antibody levels. These results will impact modeling needed for determining herd immunity generated by natural infection or vaccination.

## Highlights

• Serum IgG developed in 95% mildly symptomatic patients by day 14 and all seroconverted by day 30.
• Serum IgM and IgA antibody responses were lower and less frequent than corresponding IgG responses.
• Serum IgM and IgA responses peaked and declined earlier in 100% of mild symptomatic individuals.
• <45% of asymptomatic infected individuals had seroconverted by day 30 post-PCR diagnosis.
• Impact on modeling of herd immunity.

## Introduction

Coronavirus disease-2019 (COVID-19), the disease caused by severe acute respiratory syndrome coronavirus 2 (SARS-CoV-2), was first identified in December 2019 in Wuhan, China (Guo et al., 2020). The World Health Organization (WHO) declared a Public Health Emergency on January 30, 2020, after COVID-19 had spread to other countries (Cascella et al., 2020) and declared COVID-19 a pandemic in March 2020 (2020, WHO, 2020). The first case of COVID-19 in Bangladesh was confirmed on March 8, 2020. As of August 27, 2020, according to the Institute of Epidemiology, Disease Control and Research (IEDCR), there have been 302,147 confirmed COVID-19 cases in Bangladesh, including 4082 related deaths for a case fatality rate of 1.38%.(Anon, 2020b)

Infection with SARS-CoV-2 can cause a range of symptoms, ranging from mild illness to severe pneumonia; symptoms of the disease include fever, cough, sore throat, myalgias, headaches, diarrhea, and anosmia. In severe cases, difficulty in breathing, need for mechanical ventilation, and death can occur (Yan et al., 2020). The virus that causes COVID-19 infects people of all ages. However, evidence to date suggests that two groups of people are at a higher risk of getting severe COVID-19 disease (Haveri et al., 2020): people over 60 years old and those with underlying medical conditions (such as cardiovascular disease, diabetes, chronic respiratory disease, cancer, and obesity).

Serological responses in COVID have been reported in moderately or severely symptomatic individuals in China (Long et al., 2020a), Europe (Haveri et al., 2020), and North America (Haveri et al., 2020, Perera et al., 2020), but it is unclear how these responses would compare to those with only mild symptoms or who are asymptomatically infected. Also, it is not clear how serological responses would compare in residents of densely packed urban areas in South Asia, including localities with large informal settlement areas. It is possible that such conditions would result in more frequent exposure to non-SARS-CoV-2 coronaviridae, and as such, immune responses to SARS-CoV-2 might be distinct in such populations. Notably, a preliminary data of the Bangladesh Government survey has shown until September 2020 that about one in 10 people in Dhaka city are COVID-19 positive and most of them are asymptomatic. Meanwhile, in another government survey conducted by IEDCR, it has been reported that over 90 percent of the confirmed cases were asymptomatic COVID-19 positive (Hasib, 2020). Such differences could impact both future seroprevalence analyses as well as vaccine immunogenicity studies, and similar differences have been noted for other infections (Leung et al., 2013, Qadri et al., 2013).

To address this, we studied IgG, IgM, and IgA immune responses in individuals infected with SARS-CoV-2 in Bangladesh who were either only mildly symptomatic or asymptomatic, and followed these individuals over the subsequent 30 days; we utilized the receptor-binding domain (RBD) of the spike protein of SARS-CoV-2 as an antigen for the detection of immune responses. The novel coronavirus SARS-CoV-2 has four main structural proteins, the spike (S), envelope (E), membrane (M), and nucleocapsid (N) proteins (Du et al., 2016, Wrapp et al., 2020). The S protein helps viral entry into host cells by first binding to a host receptor through the RBD in the S1 subunit and then fusing the viral and host membranes through the S2 subunit (Li et al., 2005, Liu et al., 2004). Antibodies binding to the spike (S) protein RBD can neutralize SARS-CoV-2 (Stadlbauer et al., 2020). To better understand the utility of antibody testing not only as a diagnostic test for individuals with mildly symptomatic illness, but also as a tool for detecting subclinical infection, and to inform potential seroprevalence analyses, we assessed responses in individuals with mildly symptomatic and asymptomatic infection. Mildly symptomatic and asymptomatic infections may account for the majority of global infections in humans. To determine the specificity of the immune responses seen in infected individuals, we also measured immune responses in a group of controls, both healthy subjects as well as specimens from surveillance platforms for viruses commonly circulating in Bangladesh, e.g. influenza A and Japanese encephalitis as pre-pandemic controls.

## Methods

### Study groups and recruitment

We enrolled patients with COVID-19 infection, with or without symptoms, from in and around Dhaka using the surveillance and outbreak platform of the IEDCR. Patients with COVID-19 infection, who were confirmed by RT-PCR of nasopharyngeal or oropharyngeal swabs were recruited for this study. Asymptomatic individuals were detected either in screening programs or through contact tracing. We collected serial blood specimens from infected patients on days 1, 7, 14, and 30 following the confirmation of infection, either in the clinic or during home visits.

We included a variety of controls to determine cross-reactivity to the SARS-CoV-2 antigen in the enzyme-linked immunosorbent assay (ELISA). These included 152 single time-point plasma specimens from healthy controls (n = 73), patients with influenza A (n = 59), or specimens from the Japanese encephalitis (JE) surveillance platform (n = 20) (collected between January 2015 and November 2019 in previous studies prior to the current pandemic). All specimens from COVID-19 patients were collected through public health surveillance conducted by the IEDCR as part of the country’s response to the disease. WHO guidelines were followed to categorize illness severity (Organization, 2020). The study was also approved by the IRB of the IEDCR and icddr,b.

### Real-time polymerase chain reaction (RT-PCR)

SARS-CoV-2 RNA was detected in nasopharyngeal/oropharyngeal swabs of patients by RT-PCR using Nucleic Acid Diagnostic Kits (PCR- Fluorescence Probing), supplied by the Directorate General of Health Services to IEDCR and performed as per the manufacturer’s instructions. The classification of patients as symptomatic versus asymptomatic was determined by the presence or the absence of symptoms consistent with COVID-19 (fever, cough, weakness, headache, sore throat, vomiting, diarrhea, myalgias, and anosmia) at the time of RT-PCR testing. Symptoms were documented by phone calls and also by home visits during follow up days. Asymptomatic patients did not have any symptoms at the time of collection of the nasal swab nor in the 30 days after collection; data were not available on the symptoms in the 1–2 months prior to testing.

### Collection of blood and separation of serum

Blood (5 ml) was collected initially 2–3 days after individuals tested positive and this was designated as study day 1. In addition, blood was collected on study days 7, 14, and 30. Blood was transported to the laboratory at 2 °C–8 °C. Vacutainers were centrifuged at 700× *g* for 15 min. Serum was separated from blood and kept frozen (−80O C) until the time of laboratory analysis.

### Receptor-binding domain (RBD) protein ELISA

The RBD component is a part of the spike protein-binding domain of SARS-CoV-2, which binds specifically to the cell receptor ACE2 (Lan et al., 2020). The SARS-CoV-2 RBD was expressed in Expi293F cells with a C-terminal SBP-His8X tag and purified using affinity chromatography and then size exclusion chromatography as described previously (Norman et al., 2020). SARS-CoV-2 RBD was supplied by Dr. Aaron Schmidt (Ragon Institute MGH, MIT and Harvard). Antibody measurement was performed for the IgG, IgM, and IgA isotypes against RBD using standardized ELISA protocols (Qadri et al., 1997a, Qadri et al., 1997b) in the EPOC microplate reader (BioTek Instrument, Germany). The recombinant RBD-based spike antigen is being used for the study of immune responses in COVID-19 patients (Perera et al., 2020, Premkumar et al., 2020). In brief, 96-well Maxisorp plates (Nunc, USA) were coated with RBD antigen (1 μg/mL) in carbonate buffer (10 mM and pH 9.6) for 30 min. After washing, plates were blocked with 5% BSA in PBS and then two-fold serially diluted serum specimens starting at 1:20 for IgA and IgM and 1:50 for IgG were added to the wells for 30 min in a shaking incubator (Gallenkamp; Germany) at 37O C. Plates were developed after adding secondary anti-IgA (1:5,000), anti-IgG (1:10,000), and anti-IgM (1:10,000) antibodies (Jackson Immunoresearch, USA) and substrate (O-phenylenediamine, Sigma–Aldrich, USA), and optical densities were measured at 492 nm after stopping the reaction with 1M sulfuric acid. Repeat ELISAs were carried out at higher dilutions of serum when end-point titers could not be estimated. Antibody titers were determined by software (Gen5) using various cut offs of OD units to maximize the AUC of the assay as a positive response (see below), and the highest dilution giving a positive response expressed as an ELISA titer as done previously (Qadri et al., 1997b). We included an inhouse pooled convalescent serum prepared from COVID-19 patients that had high IgG titers to RBD on all ELISA plates for maintaining quality control to assess for any day to day variation in the assay. We designated a titer of 1 to indicate the lower limit of detection.

### Statistical analysis

The distribution of antibody responses in symptomatic and asymptomatic cases was compared by the Wilcoxon’s rank-sum test. The accuracy of the RBD ELISA was assessed by receiver-operating characteristic area under the curve (AUC) analyses. All analyses were performed using the GraphPad Prism 6.0 and STATA software version 15.

## Results

### Demographics of the study groups

In this study, we analyzed data from 171 patients positive for SARS-CoV-2 by RT-PCR. Based on WHO criteria, our data set contains only mildly symptomatic patients (n = 108) and asymptomatic individuals (n = 63). Of the 108 symptomatic patients, none met criteria for moderate or severe disease (Organization, 2020) (e.g., documented pneumonia, and oxygen saturation below 90% or required supplemental oxygen, intubation, or ICU level care) and none were hospitalized. The number of symptomatic patients with serum collected at different time points were day 1: n = 74, day 7: n = 76, day 14: n = 47, and day 30: n = 71; we obtained blood from 32 patients at all four study time points of follow up. The demographics and characteristics of the study participants are presented in Table 1.

**Table 1:** Demographic and clinical features of patients.

Characteristics	Symptomatic patients	Asymptomatic patients	Pre-pandemic controls
Number of study participants	108	63	152
Healthy controls	–	–	73
Influenza patients	–	–	59
JE platform	–	–	20
Median age in years (Min, Max)	38 (1.5, 77)	33 (15, 90)	
Healthy controls	–	–	38 (20, 49)
Influenza patients	–	–	50 (22, 80)
JE platform	–	–	3 (0.9, 98)

Gender			
Male	74 (69%)	40 (63%)	–
Healthy controls	–	–	49 (67%)
Influenza patients	–	–	46 (78%)
JE platform	–	–	11 (55%)
Female	34 (31%)	23 (37%)	–
Healthy controls	–	–	24 (21%)
Influenza patients	–	–	13 (22%)
JE platform	–	–	9 (45%)

Symptoms in symptomatic patients			
Fever	82 (76%)	–	–
Cough	75 (69%)	–	–
Sore Throat	51 (47%)	–	–
Weakness	48 (44%)	–	–
Headache	38 (35%)	–	–
Respiratory Distress	17 (16%)	–	–
Diarrhea	11 (10%)	–	–
Myalgias	9 (8%)	–	–

For the 63 people who were RT-PCR positive but were asymptomatic for COVID at the time of testing and at subsequent home visits, the number of patients with serum collected at different time points were day 1, n = 28; day 7, n = 37; day 14, n = 41; and day 30, n = 48); we had blood samples from all 4 time points from 20 individuals. The asymptomatic individuals were contacts of suspected or confirmed positive cases, frontline workers such as police and related individuals referred for routine testing or staff at IEDCR tested in screening programs.

### RBD-specific responses in symptomatic, asymptomatic patients, and controls

**Figure 1:**
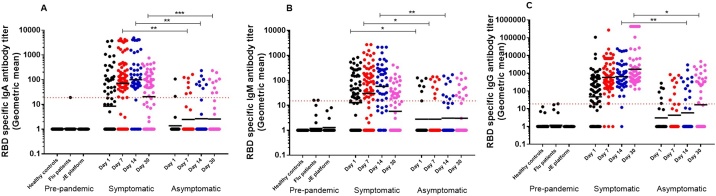
Levels of RBD-specific IgA antibodies (A), IgM antibodies (B), and IgG antibodies (C) in mildly symptomatic, asymptomatic, and pre-pandemic participants (healthy controls, patients with influenza or JE platforms). Lines represent geometric mean titers, with dots representing patients at each day (day 1: black, day 7: red, day 14: blue, and day 30: pink) after COVID-19 detection by RT-PCR. The limit of detection of the assay is set at a titer of 1. An asterisk represents a significant difference between symptomatic and asymptomatic patients between different days (* <0.05, **<0.01, and ***<0.001) using an unpaired *t*-test.

Antibody responses to the SARS-CoV-2 RBD are shown in Figure 1. We ran 73 specimens from healthy controls, 59 specimens from patients with confirmed influenza, and 20 specimens from patients from the Japanese encephalitis surveillance platform who had symptoms of acute encephalitis, which were in our collection prior to the pandemic, to determine the baseline levels of response to the SARS-CoV-2 RBD antigen in the ELISA. The large majority of the pre-pandemic healthy controls had responses to RBD below the level of detection of the assay for each isotype. For patients with influenza A, the IgA titer to SARS-CoV-2 RBD ranged from 1 to 19; IgM titers ranged from 1 to 16; and IgG titers ranged from 1 to 19. In the 20 samples from Japanese encephalitis surveillance platform, IgA and IgG titers were below the detection limit and IgM titers ranged from 1 to 8. Based on these findings, we designated cutoff values of >19, >16, and >19 for IgA, IgM, and IgG, respectively, for a positive antibody response to SARS-CoV-2 (Table 2).

**Table 2:** Specificity and Sensitivity analyses for RBD antigens against IgA, IgG, and IgM in symptomatic and asymptomatic patients.

Isotype	Cutoff	Days since PCR+	Symptomatic Patients			Asymptomatic patients		
			AUC (95% CI)	Sensitivity (%)	Specificity (%)	AUC (95% CI)	Sensitivity (%)	Specificity (%)
IgG	>19	1	0.85 (080−0.90)	64	100	0.64 (0.55−0.74)	21	100
		7	0.98 (0.96–1.00)	96	100	0.65 (0.57 – 0.73)	22	100
		14	0.99 (0.96–1.00)	95	100	0.65 (0.560 0.72)	29	100
		30	1.00 (1.00 – 1.00)	100	100	0.72 (0.65 – 0.80)	45	100
IgA	>19	1	0.87 (0.66 – 0.78)	36	100	0.55 (0.49 – 0.60)	7	100
		7	0.92 (0.88 – 0.96)	79	100	0.62 (0.55 – 0.69)	19	100
		14	0.92 (0.87 – 0.98)	83	100	0.59 (0.52 – 0.67)	20	100
		30	0.85 (0.80 – 0.91)	58	100	0.62 (0.56 – 0.68)	19	100
IgM	>16	1	0.78 (0.72 – 0.84)	51	100	0.60 (0.51 – 0.69)	21	100
		7	0.86 (0.81 – 0.91)	67	100	0.60 (0.52 0 0.67)	24	100
		14	0.93 (0.88 – 0.98)	72	100	0.61 (0.53 – 0.70)	22	100
		30	0.72 (0.66−0.79)	38	100	0.64 (0.57 – 0.71)	26	100

In mildly symptomatic individuals, IgA antibody titers to RBD were positive in 36% (95% Confidence Interval, (CI): 26% and 49%) of patients on study day 1 after the detection of infection. Seropositivity increased further on study days 7 (79%, 95% CI: 68% and 88%) and 14 (83%, 95% CI: 72% and 94%), but then declined by study day 30 (58%, 95% CI: 47% and 72%) (Table 2; Figure 1). IgM antibody titers to RBD showed a very similar pattern to IgA responses, also peaking at day 14 at 72% (95% CI: 60% and 86%) of individuals (Figure 1). IgG antibody titers to RBD were positive in 64% (95% CI: 52% and 75%) of infected individuals on study day 1, and increased on all three subsequent study days, such that 96% (95% CI: 89% and 99%), 95% (95% CI: 88% and 100%), and 100% (95% CI: 95% and 100%) of individuals were positive on study days 7, 14, and 30. (Table 2; Figure 1).

We compared immune responses in the mildly symptomatic and asymptomatic patients (Figure 1), to assess for differences in immune responses by the severity of the infection. We observed significant increases in the geometric mean titers of IgG (at day 14: 643 vs 6 and day 30: 1683 vs 18), IgM (day 1: 13 vs 3, day 7: 30 vs 3, and day 14: 57 vs 3), and IgA (day 7: 72 vs 3, day 14: 99 vs 3, and day 30: 21 vs 2) in mildly symptomatic patients as compared to asymptomatic patients. In the 32 mildly symptomatic patients from whom serial blood samples were available at 4 time points, all of them seroconverted for IgA, IgM, or IgG over the course of the follow-up period. On the other hand, fewer than 50% (9/20 with 4 serial blood samples) of the asymptomatic patients seroconverted over the course of the follow-up period.

## Discussion

SARS-CoV-2, a human beta coronavirus, is the cause of COVID-19, which was first identified in December 2019 in Wuhan, China (Guo et al., 2020). This disease rapidly spread out of China to now more than 218 countries and territories globally and was declared a pandemic by the WHO on March 11, 2020 (2020). The first case of COVID-19 in Bangladesh was confirmed on March 8, 2020 in a family contact of a traveler who had returned from Italy and presented with mild symptoms of coryza, nausea, fever, cough, weakness, and fatigue. No secondary transmission from this case was identified despite active surveillance that was carried out by the IEDCR. However, between March and June, the number of cases of COVID-19 in Bangladesh increased rapidly, and community transmission was seen. To date, there have been more than 183,795 confirmed COVID-19 cases in Bangladesh, particularly in the heavily populated capital city of Dhaka and the surrounding areas. Individuals who are asymptomatically infected and who continue to move throughout the country may be part of the reason for the rapid spread of the disease.

RT-PCR of respiratory secretions has been shown to be both sensitive and specific for the diagnosis of COVID-19 early in illness (Syrmis et al., 2004). Serological responses to the virus occur later and have been used on occasion to supplement the diagnosis in patients with negative RT-PCR results but a compatible syndrome (Zhang et al., 2020). Although serological responses have begun to be characterized in moderately or severely ill individuals in China, Europe, and the United States, it is critical to understand the performance of serological assays in patients with only mild symptoms or who are asymptomatic, as well as in dense population centers, both to attempt to determine the ratio of symptomatic to asymptomatic infection as well as to understand transmission dynamics in these areas and the levels of potentially protective immunity that may develop subsequently.

We analyzed serological results in 108 mildly symptomatic patients with COVID-19 and 63 asymptomatically infected individuals, and compared these to 152 control subjects both healthy individuals and those with documented infection with influenza A or Japanese encephalitis prior to the pandemic. We used the recombinantly produced RBD of the spike protein of SARS-CoV-2 as our antigen, as this has been used in previous immunoassays for the detection of serological responses and shown to have high sensitivity and specificity. In our study, we found a small number of patients with influenza or Japanese encephalitis who had low positive serological responses to the RBD of SARS-CoV-2. In our analysis of individuals infected with SARS-CoV-2 with mild symptoms, 72% (95% CI: 60% and 86%) symptomatically infected individuals had significantly lower response rates and titers in all three antibody isotypes.

Long et al. (2020a) recently reported serological results in 285 patients with COVID-19 in China, using a magnetic chemiluminescence enzyme immunoassay with the nucleoprotein and a peptide from the spike protein of SARS-CoV-2 as antigens. In the 63 patients in whom they had serial samples from admission until discharge, 97% seroconverted in their assay for both IgM and IgG antibodies. The median day of seroconversion after symptom onset was 13 days for both antibodies. They examined a cluster of 164 close contacts of patients with known COVID-19 and identified 16 asymptomatic individuals by RT-PCR; all had a positive serological response for IgG and IgM to the 2 antigens used. Premkumar et al. (2020) examined 77 samples at different time points from 63 patients with COVID-19 and compared immune responses to the RBD of the spike protein of SARS-CoV-2 by ELISA to 71 control patients. They found that 9 days after the onset of symptoms, 98% of patients had a positive IgG response with a specificity of 100%. Patients in their study also developed IgM and IgA antibody responses, although at somewhat lower rates. Lou et al. (2020) examined serial serum specimens from 80 patients with PCR-confirmed COVID-19 infection, utilizing a variety of ELISA and lateral flow immunoassays. Their IgM assay used RBD as the antigen and their IgG assay used the nucleoprotein of SARS-CoV-2 as the antigen. They found seroconversion rates in their population of 94% for both IgG and IgM. The median seroconversion occurred on day 10 for IgM and day 12 for IgG after symptom onset. Amanat et al. (2020) demonstrated that convalescent sera from patients with COVID-19 infection reacted strongly with both the RBD and the full-length spike protein of SARS-CoV-2, while controls with other viral infections did not.

These previous studies included mostly patients with moderate and severe symptoms of COVID-19 who were hospitalized. Our results in mildly symptomatic patients with COVID-19 in Bangladesh are similar to these previous studies, although our rates of seropositivity are slightly lower than some of these studies, particularly for IgM and IgA. This may relate to the fact that the immunoassays used by other investigators sometimes included more than one antigen and may be more sensitive than the technology used in the ELISA reported here. Nevertheless, our results are overall similar in mildly symptomatic individuals to those reported previously.

Long et al. (2020b) studied serological responses in 37 patients with asymptomatic infection with SARS-CoV-2 documented by RT-PCR, and compared those to responses in 37 symptomatic comparators matched by sex, age, and comorbidities. The asymptomatic group showed an IgG seropositivity rate of 84% 3–4 weeks following exposure, and the virus-specific IgG levels in the asymptomatic group were lower than in the symptomatic comparators. In our study, we found serological responses in asymptomatic individuals that were significantly lower than in individuals with even mild symptoms of infection. Both the Long et al. study (Long et al., 2020b) and ours used relatively small number of patients, and so it is not clear if the minor differences in the results between these studies are significant or not.

Overall, the results of this ELISA utilizing recombinant RBD of the spike protein of SARS-CoV-2 as the detecting antigen, show that the vast majority of patients with COVID-19 infection and even mild symptoms show a serological response to infection by 2 weeks or more after diagnosis. Serology may be useful in diagnosing cases suspected of COVID-19 infection, but later in the course of illness when RT-PCR may be negative, at least in upper respiratory secretions. Serology also may be useful for identifying asymptomatic infections in close contacts if RT-PCR is either not available or these individuals were exposed in the more remote past, although the sensitivity of serology in these individuals is substantially lower than in symptomatically infected individuals. Sensitive and specific serology may also be useful for estimating the prevalence of COVID-19 infection in a population to determine the level of herd immunity, either following infection in that population or vaccination.

Prospective studies will need to be done to determine the relationship between the serological responses to COVID-19 seen by ELISA and protection against subsequent infection, and how long that protective response may persist after infection or vaccination. Future studies should investigate the development of memory B cells to SARS-CoV-2 antigens following infection and whether these, in addition to or in place of circulating antibody, may mediate protection against subsequent infection, as seen in other infectious diseases (Harris et al., 2009, Leung et al., 2013, Qadri et al., 2003, Qadri et al., 1997b). Lastly, the role of the IgA in protecting against colonization of the mucosal surface should be investigated.

Our study has several limitations. We did not examine for cross-reactivity of our immunoassay with the RBD of SARS-CoV-1, MERS coronavirus or other human coronaviruses that circulate and infect humans. In the studies cited above, cross-reactivity of the RBD of SARS-CoV-2 was not seen with MERS or other human coronaviruses, but was seen with the RBD of SARS-CoV-1. Secondly, patients in our study were enrolled when they tested positive, and data on the dates of symptom onset prior to that testing were not available in the database of public health authorities. Therefore, our results are presented as days following positive testing rather than after symptom onset, as many other studies have utilized. We did not correlate our ELISA titers with neutralization activity against SARS-CoV-2, as has been done in other studies that showed that an IgG titer to RBD correlated with neutralization titers against SARS-CoV-2 (Premkumar et al., 2020). We have analyzed Ct value between mild symptomatic and asymptomatic patients for observing quantitative or qualitative differences on viral load. However, we did not observe any major difference in the level of viral load between symptomatic and asymptomatic patients in this study based on RT-PCR. Lastly, we did not have full data on whether our asymptomatic individuals had any respiratory symptoms suggestive of COVID-19 prior to their COVID-19 RT-PCR being positive, only data that they remained asymptomatic thereafter. Therefore, it is possible that the immune responses seen in our asymptomatic individuals might relate to some of those patients who have had symptomatic infection at an earlier time point.

Despite these limitations, this is the first serological study of immune responses to SARS-CoV-2 in a substantial number of mildly symptomatic and asymptomatic individuals in South Asia utilizing an easily available ELISA platform for detecting immune responses to SARS-CoV-2 infection. Our results suggest that this assay can be highly sensitive and specific for recent symptomatic SARS-CoV-2 infection. These data will be very useful for seroepidemiological studies as well as for vaccine trials when they will be rolled out in the future.

## Conflict of interest

None declared.

## Funding

10.13039/100004423World Health Organization, Fogarty International CenterTW005572, and Emerging Global Leader Award; K43 TW010362, US NIH/NIAID R01: AI130378, the Fondation Merieux, and the Bill and Melinda Gates Foundation (BMGF). This study was carried out with the support of the United States Agency for International Development (USAID)under the terms of USAID’s Alliance for Combating TB in Bangladesh: activity cooperative agreement no. CA # 72038820CA00002. Views expressed herein do not necessarily reflect the views of the US Government or USAID.
